# Exosome Structures Supported by Machine Learning Can Be Used as a Promising Diagnostic Tool

**DOI:** 10.3390/ma15227967

**Published:** 2022-11-11

**Authors:** Esra Cansever Mutlu, Mustafa Kaya, Israfil Küçük, Besim Ben-Nissan, Artemis Stamboulis

**Affiliations:** 1 College of Engineering and Physical Science, School of Metallurgy and Materials, Biomaterials Research Group, University of Birmingham, Birmingham B15 2TT, UK; 2 Department of Biomedical Engineering, Faculty of Engineering and Architecture, Beykent University, Sarıyer, 34398 İstanbul, Türkiye; 3 Institute of Nanotechnology, Gebze Technical University, 41400 Gebze, Türkiye; 4 School of Life Sciences, Translational Biomaterials and Medicine Group, University of Technology Sydney, P.O. Box 123, Broadway, NSW 2007, Australia

**Keywords:** extracellular materials, PCA, dexosomes, cryo-TEM, Fast Fourier Transform, image processing

## Abstract

Principal component analysis (PCA) as a machine-learning technique could serve in disease diagnosis and prognosis by evaluating the dynamic morphological features of exosomes via Cryo-TEM-imaging. This hypothesis was investigated after the crude isolation of similarly featured exosomes derived from the extracellular vehicles (EVs) of immature dendritic cells (IDCs) JAWSII. It is possible to identify functional molecular groups by FTIR, but the unique physical and morphological characteristics of exosomes can only be revealed by specialized imaging techniques such as cryo-TEM. On the other hand, PCA has the ability to examine the morphological features of each of these IDC-derived exosomes by considering software parameters such as various membrane projections and differences in Gaussians, Hessian, hue, and class to assess the 3D orientation, shape, size, and brightness of the isolated IDC-derived exosome structures. In addition, Brownian motions from nanoparticle tracking analysis of EV IDC-derived exosomes were also compared with EV IDC-derived exosome images collected by scanning electron microscopy and confocal microscopy. Sodium-Dodecyl-Sulphate-Polyacrylamide-Gel-Electrophoresis (SDS-PAGE) was performed to separate the protein content of the crude isolates showing that no considerable protein contamination occurred during the crude isolation technique of IDC-derived-exosomes. This is an important finding because no additional purification of these exosomes is required, making PCA analysis both valuable and novel.

## 1. Introduction

Exosomes have the potential to play an important role in applications such as drug delivery, exosome-based regenerative therapeutics, and bioprinting [1,2,3,4]. For example, in a very recent study [4], bTCP-induced macrophage exosomes were used for 3D printing to construct porous scaffolds benefiting from the ability of exosomes to enable intracellular communication. The scaffolds exhibited a predefined structure with the continuous release of exosomes and displayed immunomodulatory properties and improved osteogenesis/angiogenesis. Recent studies also showed that exosomes can be used for diagnostic purposes [5,6]. In a very recent review, it was stated that exosomes can play a key role in the diagnosis of diseases such as neurodegenerative diseases such as Alzheimer’s. Specifically, the concentrations of biomarkers and the number, size, and morphology of exosomes can also influence the differential diagnosis of early-onset neurodegenerative diseases [7]. Recent technology based on nanotheranostics, nano-magnetic, and quantum nanoparticles has shown particularly good results for the differential diagnosis of diseases such as pancreatic and Alzheimer’s disease; however, exosomes are very promising biocompatible alternative biomaterials that can provide morphological information related to specific diseases [8,9,10,11].

Einstein questioned the application of the classical chaos theory, fundamental to many subjects including biology and quantum mechanics [12]. As a result, the deterministic chaos theory was utilized to express the randomness of chaotic complex systems. The deterministic chaos theory defined random states of disorder and irregular conditions such as repetition and self-organization, as suggested by Edward Lorenz in 1963. The chaos theory states that small differences in initial conditions could cause many different results in dynamic systems, explaining their development [13]. Today’s biological big-data science includes the evaluation of organ systems, circadian rhythm, vascular biology, brain-phase transitions, and chaotic motifs in gene regulatory networks as examples of a biological chaotic environment and dynamic systems. In fact, the desired and prominent status of every dynamic chaotic system is to maintain the relationships among its components.

Likewise, exosomes in Extracellular Vehicles (EVs) represent a dynamic system in which their movements could expand beyond the vertical and horizontal dimensions. Furthermore, using image processing, the structure of exosomes and exosome sub-population interactions can be used for diagnostic purposes. A significant challenge, however, is the rigorous process of standardization during the isolation process [14,15]. Either density- or sized-based separation techniques are normally used. However, current standard separation techniques often lead to the loss of exosome subpopulations containing vital information such as exosome RNAs [16]. In addition, all these commercial separation techniques are both expensive and time consuming.

Image-processing techniques have been extensively used for diagnostic purposes. Machine learning can be used effectively to identify high- and low-frequency morphological features without the need to use other chemical separation techniques.

In this work, we questioned whether morphological features from isolated exosomes could be promising to identify changes in cells and use them as disease prognosis and/or diagnostic tools. For this reason, exosomes were isolated from immature dendritic cells (IDCs), and all structures were characterized by FT-IR and Cryo-TEM. The impurities were evaluated using both SEM and SDS-PAGE. Brownian Motion principal nanoparticle tracking analysis (NTA) results representing a stochastic approach were compared with a chaotic evaluation approach using Cryo-TEM images and Principal Component Analysis (PCA).

## 2. Materials and Methods

### 2.1. IDC Production and Isolation

IDCs were produced and isolated according to previous work by Mutlu et al. [17]. Phosphate-buffered saline (PBS) was produced in the lab, and fetal bovine serum (FBS) was supplied by VWR. PBS, double-distilled water, and FBS were ultra-centrifuged overnight at 120,000× *g* and the supernatant was collected.

In addition, the immature dendritic cell line, JAWS II, (CRL-1194; ATCC) was grown in DMEM-F12 full-growth medium. The medium was supplemented with 10% FBS, 4 mM L-glutamine, 1% penicillin-streptomycin, and 25 ng/mL murine GM-CSF at 37 °C in a 5% CO2 environment. The cell culture passages were performed by separating non-adherent and adherent cells after treatment with 0.25% trypsin-0.03% EDTA (supplied by Gibco). Subsequently, the cells were transferred into the new cell culture flasks. Prior to performing the exosomes’ isolation, healthy cells were counted using an automated TC20TM cell counter (Bio-Rad, Hercules, CA, USA). Exosomes from non-adherent IDCs were centrifuged at 300× *g* for 15 min at 4 °C. Then, the cell pellets were removed, and the supernatants were gently poured into 8 mL ultra-centrifuge tubes on dry ice. The ultra-centrifugation process was performed according to previous work by Mutlu et al. [17].

### 2.2. Nanoparticle Tracking Analysis (NTA)

The Nanoparticle Tracking Analysis was performed using the Malvern© NTA NS300 to evaluate the size and concentration of exosomes. Tracking video records of exosomes were also obtained. The exosomes (3 vials × 5 μL) received from the purification, as described above, were then diluted 10×, and the concentration of the exosomes was determined. The video recordings were analyzed by the FIJI-ImageJ program™ using the TrackMate Plugin [18].

### 2.3. SDS-PAGE Gel Electrophoresis

Sodium dodecyl sulfate-polyacrylamide gel electrophoresis (SDS-PAGE) experiments were performed using 12% (*w*/*v*) polyacrylamide gels containing 0.1% (*w*/*v*) SDS, as described in the study conducted by Ossipow et al. to confirm the production of the wild-type and mutant BSH enzymes. The protein bands were detected using Coomassie Brilliant Blue R250 staining [19]. A PageRuler™ Prestained Protein Ladder, 10 to 180 kDa, supplied by ThermoFisher was used as a size standard for SDS-PAGE.

### 2.4. Scanning Electron Microscopy (SEM) of Exosomes

SEM studies were performed by the Zeiss—Evo|MA10© Scanning Electron Microscope (Jena, Germany) to explore the surface characteristics of exosomes. Twenty-five microliter samples in exofree-PBS were placed on an SEM grid by using a Pasteur pipette, and the room temperature was set at 17 °C. The SEM sample was coated with gold (~500 × 10^−12^ μm in thickness) using a sputter coater under a high vacuum. The surface features of the samples were observed at a magnification of 20× to 200× and the images were evaluated and analyzed by Fiji-ImageJ™ Software (ImageJ bundled with Zulu OpenJDK 13.0.6).

### 2.5. FT-IR Analysis of Exosomes

The crude samples placed in exofree-PBS were lyophilized in a sterilized environment and, subsequently, the dried powders were examined under a Shimadzu 8400S FTIR spectrophotometer (Kyoto, Japan). The measurements were taken between 400 and 4000 cm^−1^ in both transmission and absorbance modes, respectively.

### 2.6. Cryo-Transmission Electron Microscopy (Cryo-TEM) Studies of Exosomes

The sample preparation was created with a GP Plunge Vitrification Robot. For this purpose, a copper 200 mesh quantifoil TEM grid with 10 uL of the sample was placed in the vitrification robot at the appropriate position with the help of tweezers. The sample was vitrified using liquid nitrogen. The vitrified sample was examined under a Hitachi HT 7800 brand TEM microscope (Tokyo, Japan) below −178 °C. The cryo-TEM images were analyzed using Fiji-ImageJ [20].

### 2.7. Three-Dimensional (3D) Image Processing of Exosomes

The experiments in this section were performed as prescribed according to Mutlu et al. [6]. The CellMask™ Green Plasma Membrane Stain was applied to follow the lipid structures. After the small T-flask production, the isolation procedure was performed, and confocal microscopy studies were performed rapidly under low and high magnification at 488 nm. Before the observation, the centrifugation of the samples was performed for 10 min at 4000× *g* using Amicon^TM^ Ultra-2 filters to discard the excess dye followed by an additional centrifugation for 2 min at 1000× *g* and a reverse spin [6]. The confocal microscopy images were converted to 3D images using Fiji- ImageJ™ Software (ImageJ bundled with Zulu OpenJDK 13.0.6).

## 3. Results

**Figure 1:**
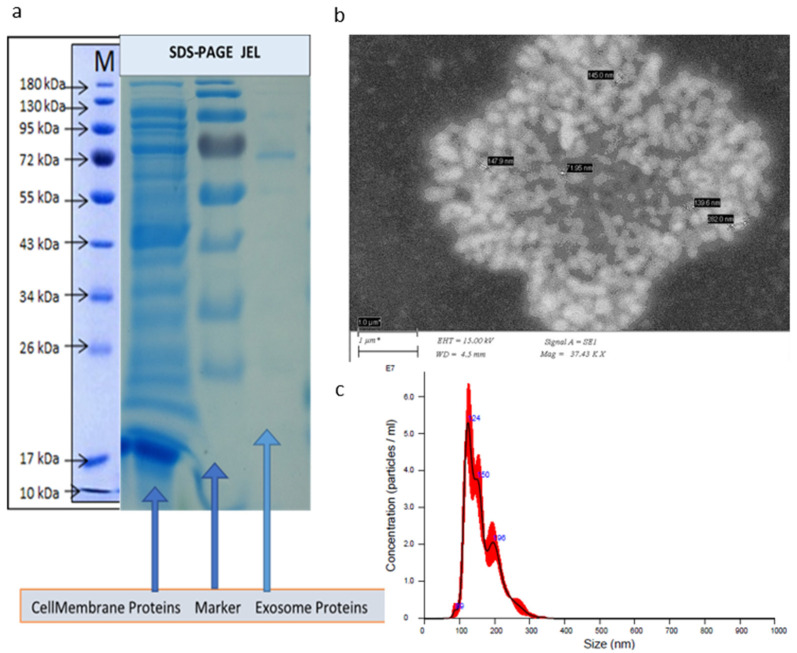
(**a**) SDS-PAGE gel results recorded for surface proteins of the IDC-derived exosomes displayed a higher molecular weight (MW) value of 72 kDa. The MW of membrane proteins varied between 10 kDa and 180 kDa; (**b**) an SEM image of the IDC-derived exosomes; (**c**) NTA data showing the size distribution of IDC-derived exosomes. *: Video S1 and Figure S1.

SDS-PAGE electrophoresis, shown in Figure 1a, helped to identify the surface proteins of IDC-derived exosomes and their profiles. The proteins of the IDC-derived exosomes had a molecular weight of 72 kDa. Other protein bands (for example, 95 kDa and 130 kDa) can also be observed, but they are present in very small amounts. The above observation can prove that exosomes could be isolated without impurities. Xun et al. [21] followed a similar crude isolation protocol successfully to isolate exosomes derived from olfactory mucosa mesenchymal stem cells (OM-MSC) without impurities. SEM images were obtained immediately after the exosomes’ isolation and are shown in Figure 1b. It is quite difficult to obtain SEM images from isolated exosomes as the IDC-derived exosomes were quite mobile. We succeeded in obtaining an image of isolated IDC-derived exosomes at high magnification (50,000×). At such a high magnification, the IDC-derived exosome structures vibrated, and their shape changed continuously.

According to the NTA data, the average size of the isolated IDC-derived exosomes was 161.0 ± 3.7 nm, as shown in Figure 1c, in agreement with the literature that clearly states that the size of exosomes should be in a range between 20 nm and 200 nm [17]. The NTA data represent an approach where the average size of the exosomes can be calculated without taking into consideration any interaction between the exosomes but considers individual exosomes in the X and Y displacements. In the supplementary data provided in Video S1 and Figure S1, it is clear that the Z displacement was not considerable. This is clearly a limitation of the technique. Analysis based on Brownian motions gives the results of the random movement of exosomes in any direction without considering interactions between exosomes. As seen in the NTA video recording Video S1 in the supplementary data, IDC-derived exosomes perform a “random walk”. The Brownian walk path of each single IDC-derived exosome was observed and imaged. A particle tracking method [22] was applied to visualize this Brownian walk as seen in Figure S1 of the supplementary data. X, Y, and Z displacements and positions were plotted versus time. However, it is clearly noticed that exosomes can move in all X, Y, and Z directions. It is the Z direction that needs to be examined more closely. Figure S1 of the supplementary data shows clearly that there is important information that can be gathered from the exosomes moving in the Z direction.

**Figure 2:**
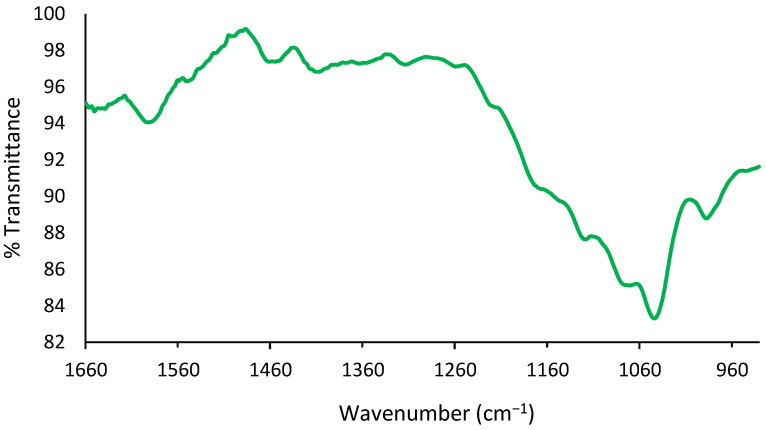
Amide I transmittance band at 1650 cm^−1^ and amide II transmittance band at 1550 cm^−1^ are observed. Aromatic ring stretching vibrations of C=C-C are observed at 1455 cm^−1^.

Zlotogorski-Hurvitz et al. reported that FTIR spectra analysis of oral cancer-cell-derived exosomes revealed that the spectra included small differences compared to the spectra taken from healthy individuals’ cell-derived exosomes, showing the potential of this technique to identify subtle changes in the spectra of exosomes using a combination of LDA-PCA machine learning techniques [23]. Recently, Uthamacumaran et al. reported a Raman spectroscopy study using extracellular vesicles derived from cancer patients’ blood samples. The authors studied colorectal cancer, hepatocellular carcinoma, breast cancer, and pancreatic cancer in combination with a machine intelligence-assisted early cancer screening approach [24]. Another pilot study performed by Martins et al. in 2020 used multivariate data analysis [25] and suggested that both Fourier Transform Infrared (FTIR) spectroscopy of blood exosomes and cerebrospinal fluid (CSF) biomarkers of Alzheimer patients’ blood samples could be significant for the successful diagnosis of the disease. The main question here is whether FT-IR is accurate enough to be used for the evaluation and comparison of IDC-derived exosomes. FT-IR spectra of the crude isolated IDC exosomes show strong peaks at 1650 and 1659 cm^−1^ associated with the presence of amide linkages from fatty acyl groups, as shown in Figure 2.

**Figure 3:**
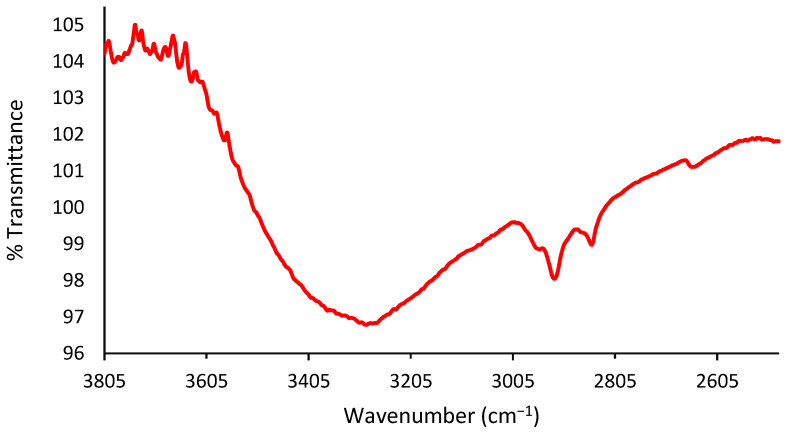
CH_2_ symmetric stretching transmittance band is observed at 2851 cm^−1^; CH_2_ asymmetric bending transmittance is observed at 2952 cm^−1^. OH- stretching vibrations band is centered at 3293 cm^−1^.

In the literature, a study conducted by Yoshida and Koike in 2011 showed similar FTIR results in exosome membrane structures [26]. Furthermore, Paolini et al. in 2020 reported that EVs show an amide I absorption band at 1650 cm^−1^ associated with carbonyl group stretching vibrations and an amide II absorption band at 1550 cm^−1^ associated with N–H bending vibrations [27]. Furthermore, transmittance bands in the range between 2655 and 2953 cm^−1^ and the range between 1363 and 1461 cm^−1^ are valid evidence of the presence of lipid acyl chains, in agreement with the studies by Mihály et al. in 2017 and Stępien et al. in 2021 [28,29]. In addition, OH- stretching vibrations were centered at 3293 cm^−1^, while -CH stretching vibrations of unsaturated lipid chains were observed at approximately 3005 cm^−1^. Finally, CH2/CH3 stretching vibrations indicating the presence of lipids appeared in the range between 3044 and 2851 cm^−1^, as seen in Figure 3.

**Figure 4:**
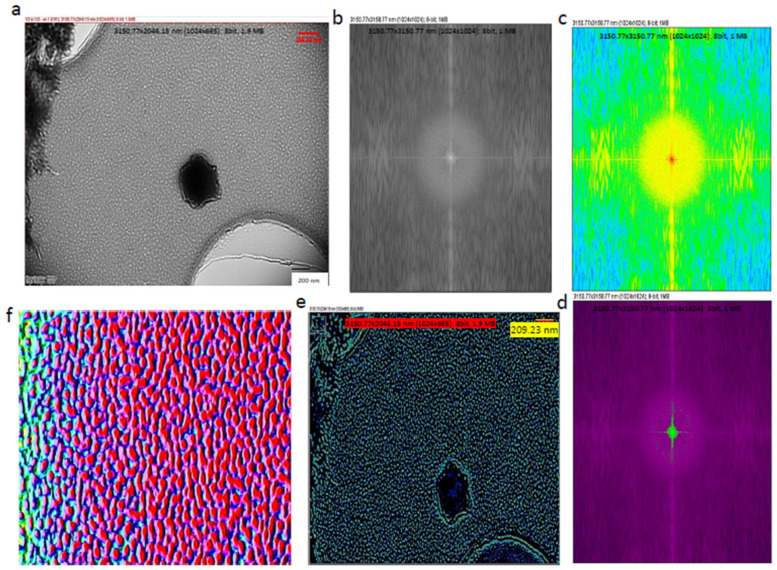
Cryo-TEM image analysis. (**a**) Cryo-TEM images of the IDC-derived exosomes; (**b**) Fourier transform image of IDC-derived exosomes; (**c**) identification of lower and similar frequencies; (**d**) identification of lowest and most prominent frequencies; (**e**) identification of specific properties of exosome components; (**f**) image converted to 3D image to identify exosomes as particles. During the conversion from 2D to 3D, all images had the same number of pixels (1024 × 1024).

Fast Fourier Transform (FFT) can preserve the information in the original data and, therefore, this method is capable of transforming images between the spatial and frequency domains [30]. This also can be expressed as Fourier analysis, converting a signal from its original domain (time or space) into the frequency domain; on the other hand, its inverse Fourier Transform leads to the decomposition of a sequence of values into components of different frequencies. In order to increase precision and accuracy, Fourier algorithms were mostly used, leading to detailed images. Cryo-TEM studies were also performed and are presented in Figure 4a; subsequently, FFT conversions were applied in low-frequency regions in order to find key differences among the exosomes [31]. Even the interior and outer layers of the exosomes could be recognized clearly using Fast Fourier Transform (FFT), as seen in Figure 4b,c. During the FFT, similar frequencies were visualized with the same color, so that the lowest-frequency regions were identified, and their inverse Fourier Transform was obtained, as seen in Figure 4e. The Fiji-image J technique was used for the 3D exosome reconstruction of the images followed by machine learning (Figure 4f).

**Figure 5:**
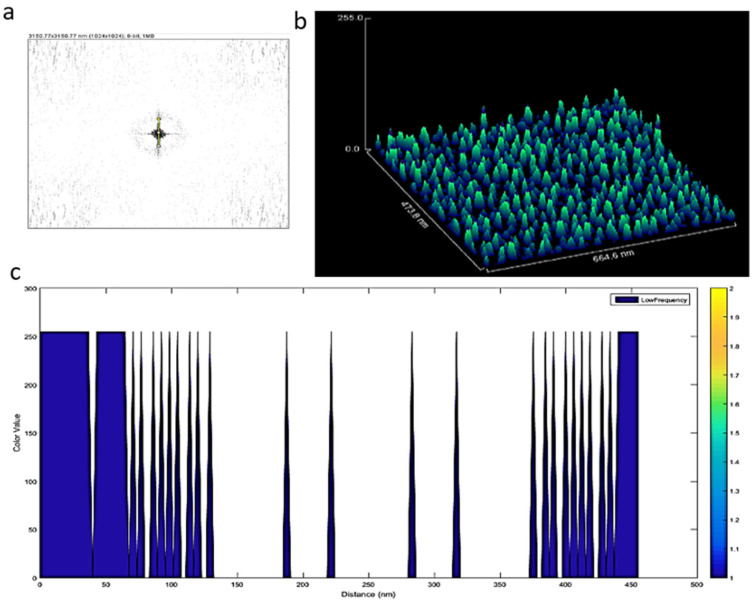
(**a**) Lowest frequency of the FFT of the Cryo-TEM images; (**b**) surface image of exosomes; (**c**) a regular distance of ca. 10 nm between exosomes was observed.

When the lowest-frequency regions were analyzed (Figure 5a), exosomes of similar dimensions were regularly distributed ~5 nm on the TEM grid (Figure 5b,c). However, confocal images showed that large particles were displayed in different shapes and sizes in the micron region within EVs (see supplementary data Video S2).

**Figure 6:**
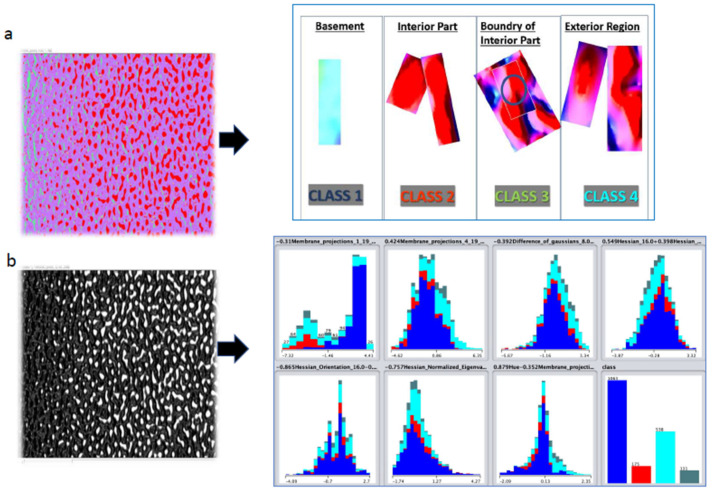
(**a**) 3D surface morphologies taken from the Cryo-TEM images. The information was used to develop a classification system; (**b**) visualization of exosomes according to their morphological features. The information was used for training the unsupervised learning.

The question that needs to be answered is whether image processing could help to find morphological differences among the exosomes. IDC exosomes have generally very similar profiles. The 3D surface images based on the Cryo-TEM images were obtained by using the Fiji-Image J software. Then, a classification system was developed as shown in Figure 6a, which was used for training the unsupervised learning. The classification system consisted of four types of IDC exosome morphologies; (a) Interspace among exosomes (Class 1 and marked as blue color), (b) regular shape of the interior part of a single exosome (Class 2 and marked as red color), (c) interior part of exosomes forming a bottleneck (Class 3 and marked as green color), and (d) shape of the exterior part of exosomes including tilted or twisted exosomes (Class 4 and marked as cyan color), as seen in Figure 6a. Initially, black-box unsupervised machine learning was performed. The following filters were used: Membrane projections, differences in Gaussian and Hessian, Hessian normalized eigenvalue, Hessian orientation, hue, and class were applied to evaluate the 3D orientation, shape, size, and brightness of the dynamic exosome morphologies (see Figure 6b).

**Figure 7:**
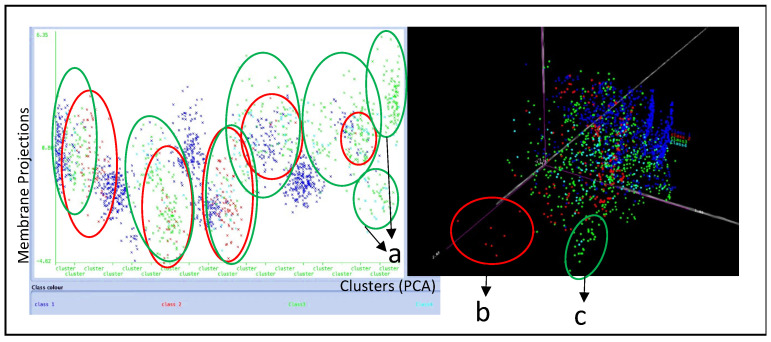
(a) Membrane projections of 19 different clusters: 14 of the clusters were dominated by class 3 exosomes, which are more mobile and dynamic. Seven different static exosomes; (b,c) distinct dynamic exosome morphologies were noticed using PCA.

Then, principal component analysis (PCA) was performed. The results of the analysis showed that the IDC-derived exosomes’ morphology appeared to be broad and diverse. Similar morphological features were identified, such as edges and convex structures, ridges, vascular spaces, and gradient changes among exosome membrane projections in X, Y, and Z directions. All these morphological features were grouped into 19 clusters of exosomes, as presented in Figure 7. Five of the clusters mostly consisted of Class 1 exosome morphologies (marked as blue). On the other hand, it is also clear that more static exosomes of Class 2 (marked as red) appeared to cluster in the presence of more dynamic exosomes of Class 3 (marked in green). Notably, clusters 18 and 19 consisted of morphologically different dynamic exosomes (Class 3 marked as green) shown in Figure 7a. The three-dimensional PCA analysis, however, showed a cluster of exosomes belonging to Class 2 (static exosomes marked as red) shown in Figure 7b. Another cluster of dynamic exosomes of Class 3 marked in green was also noticed as a separate subpopulation, as shown in Figure 7c. In addition, a population of cyan-colored exosomes belonging to Class 4 was distributed and clustered randomly together with Class 1, 2, and 3 exosomes.

## 4. Discussion

Exosomes of EVs have been recently introduced as promising therapeutic and diagnostic components in Materials Science and Engineering [32,33,34,35,36]. Green et al. reported that exosomes of EVs are recognized as miniature protocells that, if programmed and controlled, could be used for complex tissue-engineering possibilities. Protocells are able to self-assemble within synthetic tissues and hybridize with natural cells in an evolutionary process suggesting the potential of exosomes to be used in a similar role [37]. Zhang et al. in 2021 reported that extracellular nanoparticles derived from exosomes, called supermeres, can be defined as disease biomarkers and used easily for diagnostic purposes, possessing both a unique morphology and RNA content [38]. The remarkable exosome diagnostic potential was first introduced by the study of Zlotogorski-Hurvitz et al. in 2019, which reported early oral cancer diagnosis using information from saliva-derived exosomes. The absorbance spectra of the FTIR of these exosomes were evaluated according to a PCA–linear discriminant analysis with vector machine (SVM) classification and K-fold cross-validation [23,39]. For the purpose of this paper, easy single-step crude exosome isolation was used according to Mutlu et al. to avoid any potential loss of data during the isolation procedure [17].

In the near future, it is expected that deep learning techniques will provide information based on exosomes derived from patients’ cells. Both the EVmiRNA database and the EV-TRACK crowdsourcing knowledge base will benefit from deep-learning techniques to enrich the exosome bioinformation [40,41]. The EVmiRNA database can link concerned miRNAs to related metabolic pathways owing to disease paths as the evaluation of the system biology. In addition, EV-TRACK will then be able to identify differences in methodologies and validate them. Label-free diagnosis is better, even if standardization is a potential problem during any exosome research study when crude exosome isolation is used. Any further purification could lead to the loss of important information [42,43]. Lee et al. reported that data augmentation can resolve the sample deficiency of exosomes. Raman spectra data can be used by a convolutional neural network (CNN) in order to evaluate vibrations of four different EV subtypes derived from blood products, such as red blood cells and platelets, prostate cancer cell line PC3, and lymph node carcinoma of the prostate LNCaP. For the training of the neural network, 300 original data and 900 added random noise data were used for the data augmentation of four EV subtypes. The data obtained had better classification accuracy of >90% compared with the PCA. In this paper, however, it was not possible to apply image data augmentation due to the limited number of IDC-derived exosome samples and the available cryo-TEM images. Despite the above limitations, the achieved PCA accuracy was >96% [44]. The Random-Forest classification technique was applied, and results showed that the IDC-derived exosome clusters were highly different from each other, although they were derived from immature dendritic cells of similar morphology. Our study shows clearly that avoiding data loss during exosome purification is more important than using data augmentation methods.

Shin et al. used PCA with a deep learning technique to analyze data from surface-enhanced Raman spectroscopy (SERS) of lung cancer exosomes derived from human plasma samples of 43 patients. The isolated exosomes were further concentrated by filtering using a 100-kDa Amicon^TM^ filter. Although they used the Adam optimizer, it was clear that they still needed a larger amount of data—possibly lost during the additional filtering performed—to improve the accuracy of the analysis [45]. Another study by Uthamacumaran et al. was performed with exosome samples from just nine patients consisting of four different cancer subtypes using FTIR as the complementary approach. They reached high accuracy with liquid biopsy samples with iodixanol separation after crude isolation by using artificial intelligence for early cancer screening. They then concluded that Raman spectra constituted a better technique to improve the accuracy of the study compared to FTIR [24]. Our study, however, proved that even the PCA analysis of limited exosome cryo-TEM images revealed the differences from the FTIR spectra without the need for image data augmentation for training. Exosomes have great morphological diversity. We believe that cryo-TEM has exciting potential for the diagnosis and prognosis of diseases even if exosome structures and motion profiles are very similar to each other. As long as enough exosome samples are available, FT-IR spectroscopy can be used as a complementary technique to image analysis; however, cryo-TEM-derived data have greater potential to be used in deep learning techniques such as image processing and, certainly, for the evaluation and interpretation of the data.

## 5. Conclusions

Understanding the morphological differences of IDC-derived exosomes with the utilization of a machine learning technique such as PCA consists of an interesting approach to realize the range of the promising diagnostic potential of the exosome’s dynamic structures. The IDC-derived exosomes do not require further purification after their crude isolation is performed. By contrast, it is necessary to preserve all the morphological characteristics as they carry essential information that can be used to identify crucial changes that, with the help of machine learning, can become a useful tool for disease diagnosis and prognosis. The combination of image processing and machine learning can be a useful resource of morphological features for a label- and assay-free disease diagnosis.

## Data Availability

Not applicable.

## Supplementary Materials

The following supporting information can be downloaded at: https://www.mdpi.com/article/10.3390/ma15227967/s1.
