# Supplementary material for: Exosome Structures Supported by Machine Learning Can Be Used as a Promising Diagnostic Tool
Source: Materials (Basel). 2022 Nov 11;15(22):7967. doi: 10.3390/ma15227967 (PMC9693854; doi:10.3390/ma15227967)
Supplement: Supplementary file 1 [file materials-15-07967-s001.zip › Figure S1.pdf]

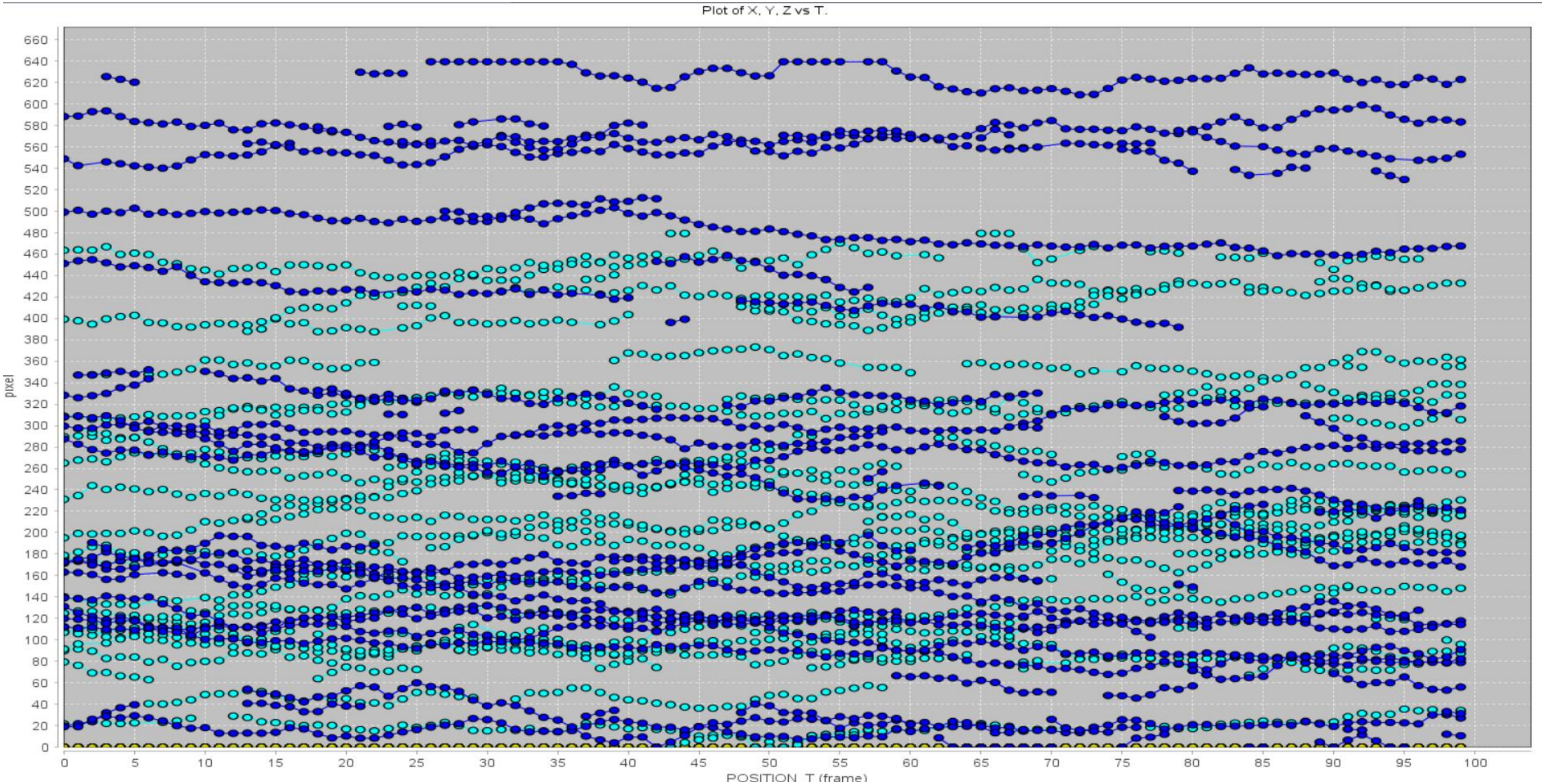

Plot of X, Y, Z vs T.
pixel
POSITION_T (frame)

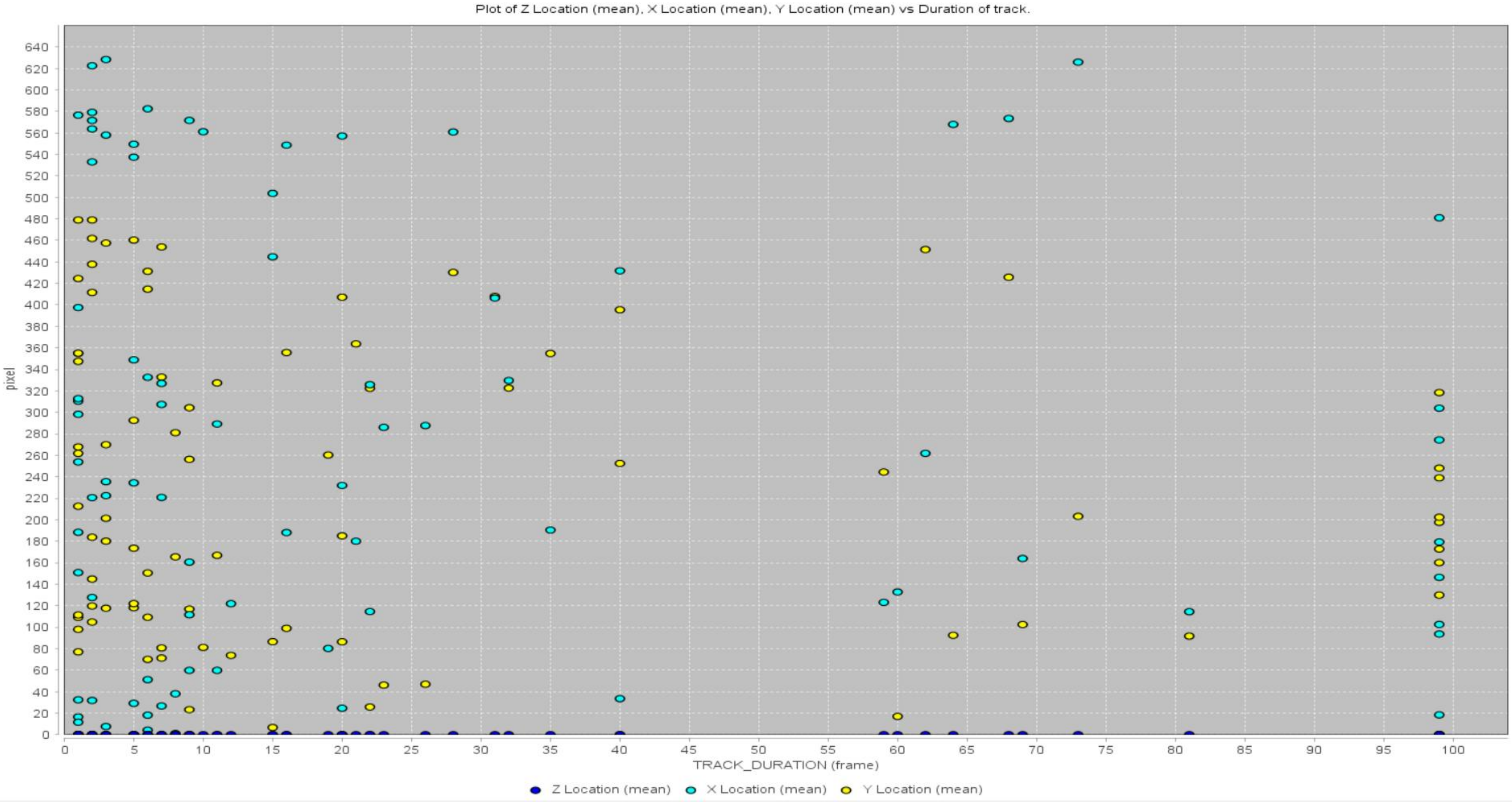

Plot of Z Location (mean), X Location (mean), Y Location (mean) vs Duration of track.
pixel
0
20
40
60
80
100
120
140
160
180
200
220
240
260
280
300
320
340
360
380
400
420
440
460
480
500
520
540
560
580
600
620
640
0
5
10
15
20
25
30
35
40
45
50
55
60
65
70
75
80
85
90
95
100
TRACK_DURATION (frame)
Z Location (mean)
X Location (mean)
Y Location (mean)

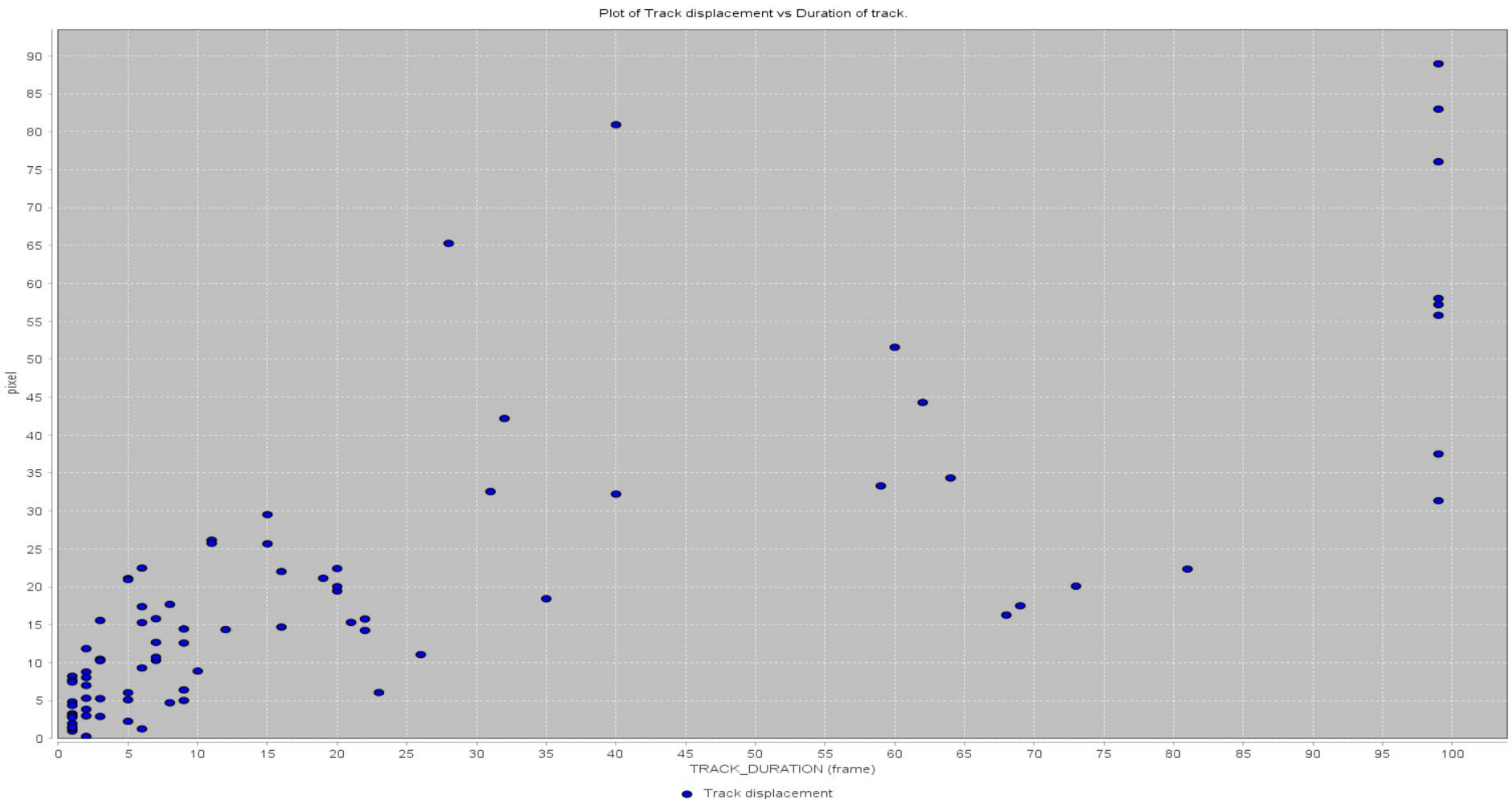
Plot of Track displacement vs Duration of track.
pixel
TRACK_DURATION (frame)
Track displacement
0
5
10
15
20
25
30
35
40
45
50
55
60
65
70
75
80
85
90
95
100

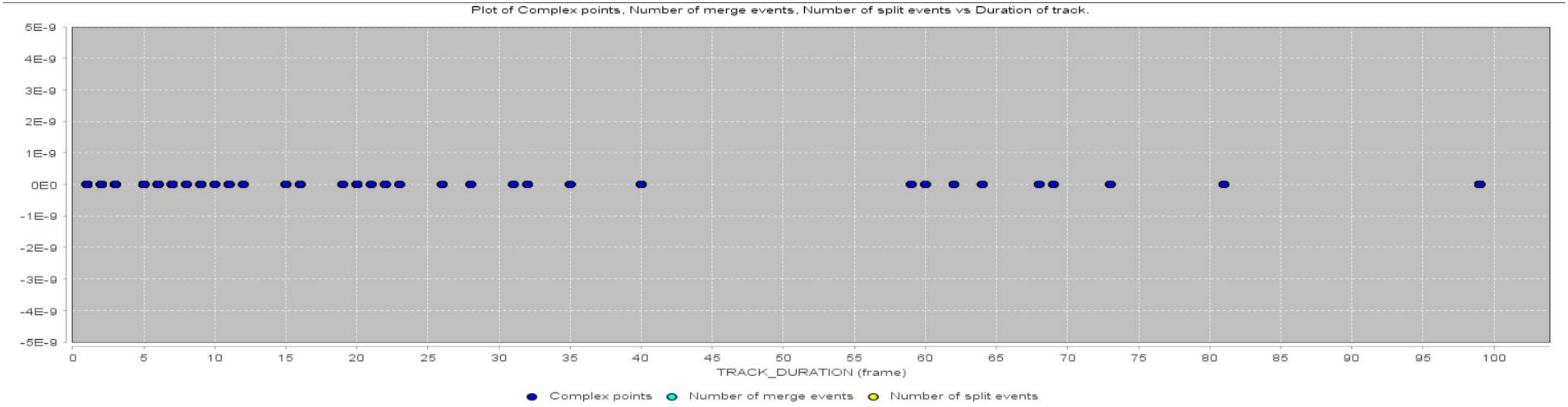

Plot of Complex points, Number of merge events, Number of split events vs Duration of track.
TRACK_DURATION (frame)
Complex points
Number of merge events
Number of split events

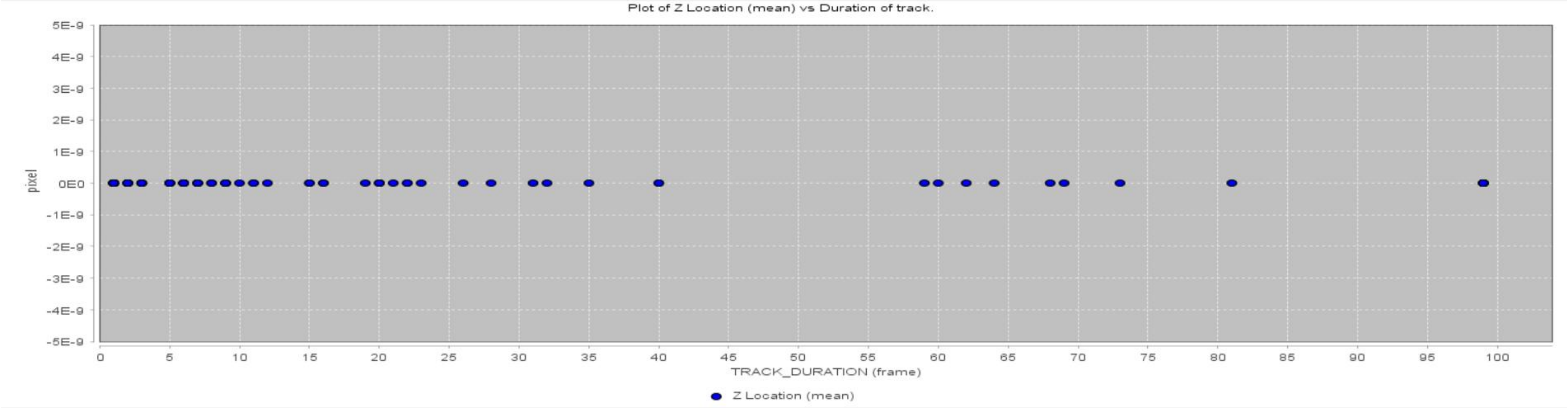

Plot of Z Location (mean) vs Duration of track.
pixel
TRACK_DURATION (frame)
Z Location (mean)

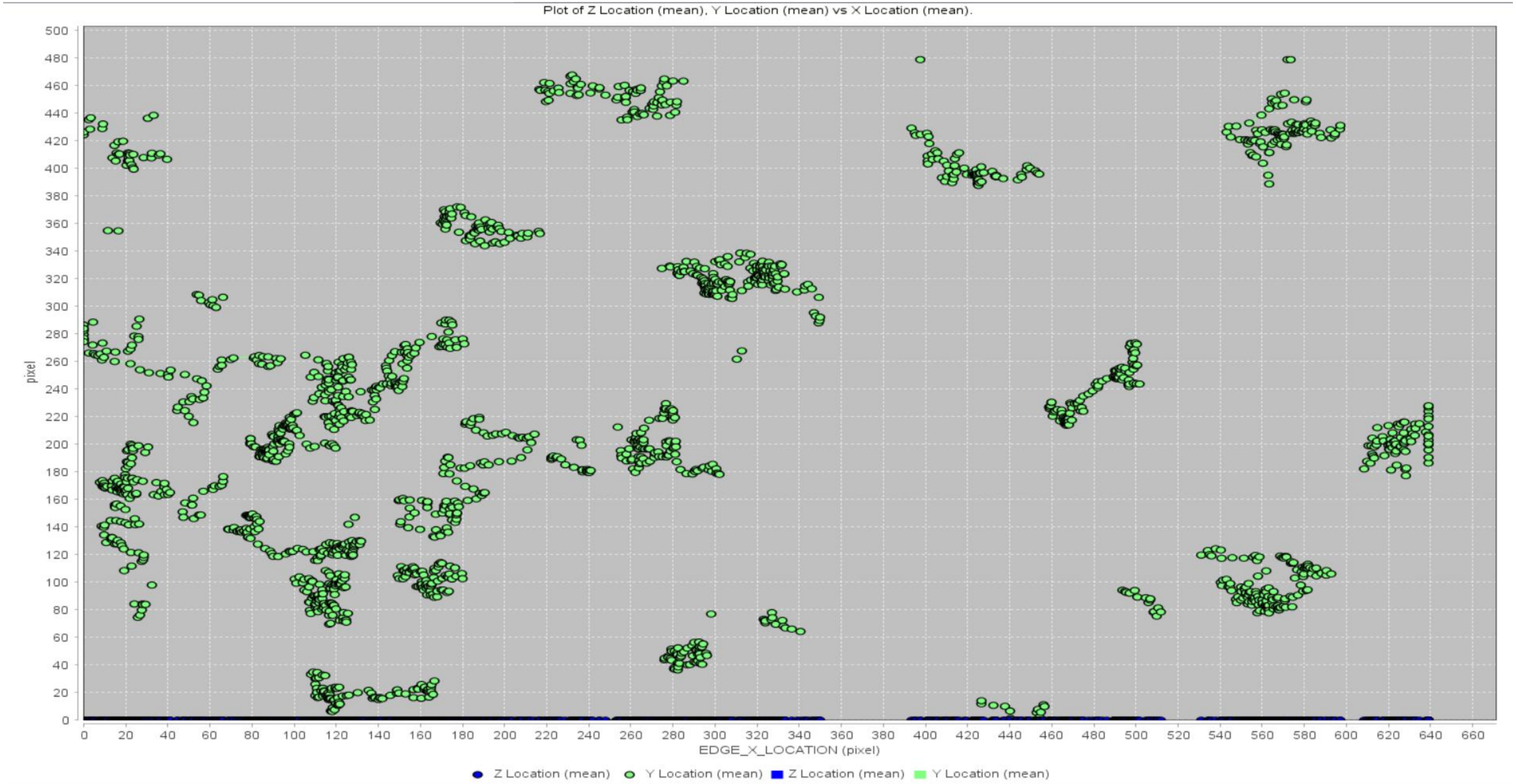
Plot of Z Location (mean), Y Location (mean) vs X Location (mean).
pixel
EDGE_X_LOCATION (pixel)
Z Location (mean)
Y Location (mean)
Z Location (mean)
Y Location (mean)

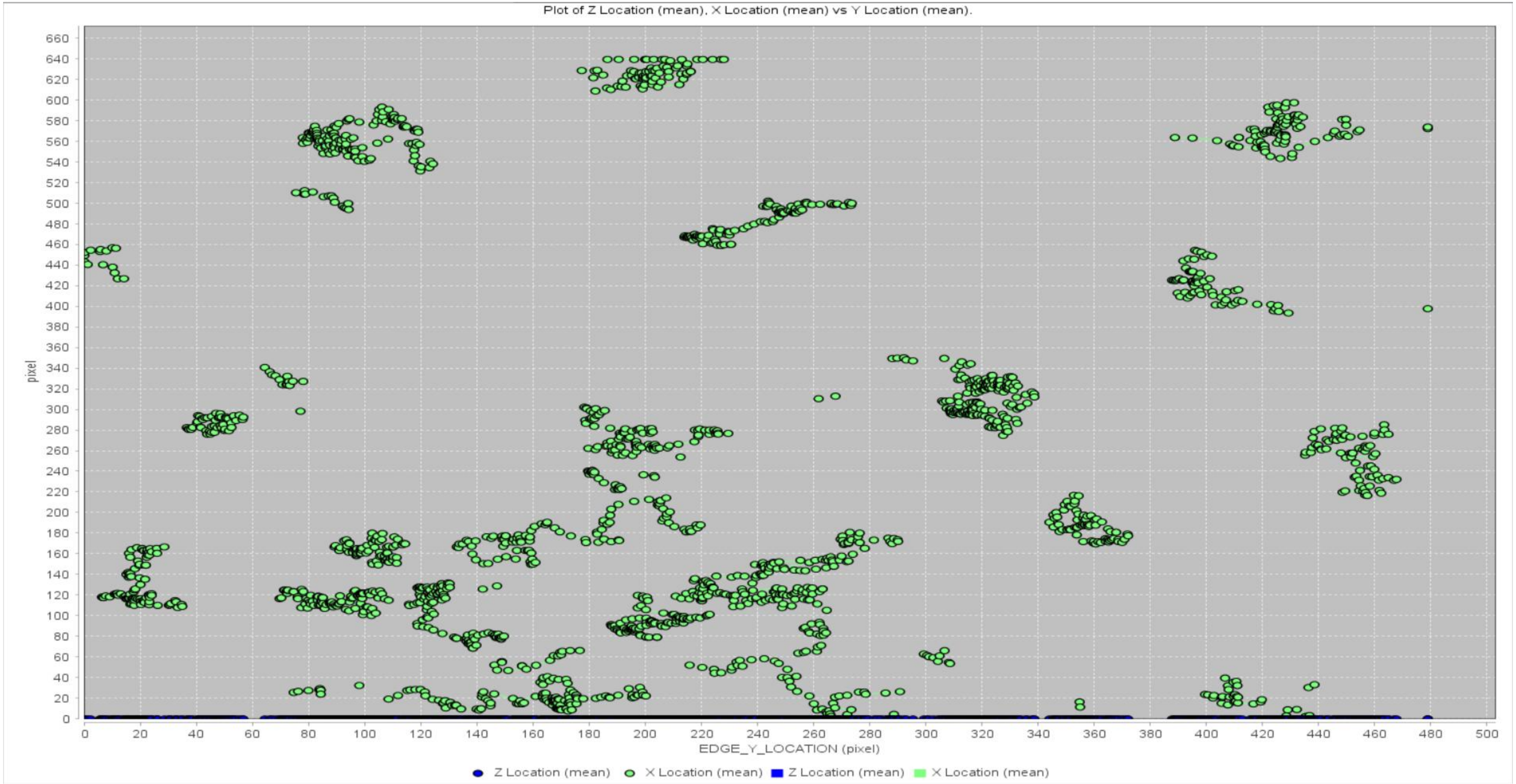

Plot of Z Location (mean), X Location (mean) vs Y Location (mean).
pixel
EDGE_Y_LOCATION (pixel)
Z Location (mean)
X Location (mean)
Z Location (mean)
X Location (mean)
